# Editorial: Rational design and preparation of nanostructured biomaterials

**DOI:** 10.3389/fchem.2022.987447

**Published:** 2022-08-15

**Authors:** Jianhua Li, Jichuan Qiu, Shaohua Ge

**Affiliations:** ^1^ Department of Biomaterials, School and Hospital of Stomatology, Cheeloo College of Medicine, Jinan, China; ^2^ Shandong Key Laboratory of Oral Tissue Regeneration, Jinan, China; ^3^ Shandong Engineering Laboratory for Dental Materials and Oral Tissue Regeneration, Jinan, China; ^4^ The Wallace H. Coulter Department of Biomedical Engineering, Atlanta, GA, United States; ^5^ State Key Laboratory of Crystal Materials, Jinan, Shandong, China; ^6^ Department of Periodontology, School and Hospital of Stomatology, Cheeloo College of Medicine, Jinan, Shandong, China

**Keywords:** biomaterials, nanostructure, tissue engineering, disease therapy, nanomedicine, stem cell

**FIGURE 1:**
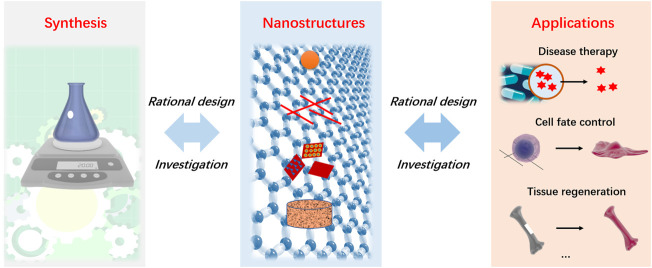
Overview of design, preparation and applications of nanostructured biomaterials.

Nanostructured biomaterials can regulate biological processes by interacting with biomolecules, cells and tissues, as they demonstrate versatile performances due to their tunable physical, chemical, and biological properties. They have encouraged significant progress for emerging biomedical fields such as tissue engineering, novel theranostic, drug delivery, antibacterial applications and so on. However, one major challenge of current biomaterial Research is the controlled regulation of biological events *via* different materials due to the limited understanding of material-bio interactions. As such, it is necessary to develop novel nanomaterials with rational design to reveal the underlying mechanisms and to meet various needs in these different biomedical applications. Over the years, with the rapid development of material science and engineering, people can solve important biomedical problems by preparing nanomaterials with versatile functionalities, pushing the Frontiers of biomaterials forward in translational and clinical settings (Figure 1). This Research Topic aims to cover recent Research development of rationally-designed nanostructured materials working for biomedical applications.

In this Research Topic, we have collected a total of 6 articles, which demonstrate the design, preparation and application of some nanostructured biomaterials such as nanowires-based scaffold and implant with novel surface nanostructures; and summarize the current progress of functional biomaterials such as nanofibers and hydrogels in exciting fields including regenerative medicine as well as disease therapy. The articles published in this Reasearch Topic are briefly summarized below.

Xue et al. reviewed rational design and preparation of functional hydrogels for skin wound healing. In this Mini Review, they introduced the design and construction of various types of hydrogels; summarized the recent advances on the functionalization of bioactive hydrogels for anti-bacteria, anti-inflammatory, tissue proliferation and remodeling purposes; highlighted the design strategies of external physical stimuli responsive hydrogels. Specifically, nano-scaled fillers could reinforce hydrogels to provide additional properties for tissue regeneration, for instance, MnO_2_ nanosheets to provide oxygen to the wound site, nanomicelles loaded with anti-inflammatory drugs to promote would healing, as well as antibacterial metal nanomaterials to treat the infection at the wound site. This review provides a comprehensive summary and future perspectives of the design and preparation of hydrogels with specific functionalities for skin would healing, which may also provide valuable implications for regenerative medicine.

For skin wound healing, it is usually difficult to be cured by conventional topical administration because it has been recognized that stratum corneum is the major obstacle for transdermal drug delivery. To address this issue, Hao et al. developed a microneedle array patch using a blend of kangfuxin (KFX), chitosan (CS), and fucoidan (FD), named KCFMN, for accelerating full-thickness wound healing. Showed that the KCFMN patch displayed noticeable antibacterial properties and good cytocompatibility. In particular, the KCFMN patch significantly accelerated the wound healing development in a full-thickness wound in rats by improving the epithelial thickness and collagen deposition. Thus, this versatile KCFMN patch has great prospects as a dressing for full-thickness wound healing.

For disease therapy, nanostructured biomaterials have offered unique advantages as well. Wu et al. reviewed the recent Research progress of electrospun nanofibers to treat various oral diseases. Oral diseases have a relatively high prevalence rate that have been recognized as huge threat to public health. In recent years, electrospun nanofibers have shown great potential in biomedical fields especially for disease treatment, because they are an ideal platform for loading and delivering bioactive molecules due to their large specific surface area, as well as regulating cell behavior as they could easily mimic the extracellular matrix. They described the engineering strategies of electrospun nanofibers; introduced the biological functions that nanofiber scaffolds could provide; and indicated future development directions in terms of scaffold design, manufacture, engineering, and clinical applications. In another review, Shi et al. summarized recent development of nanomaterials for osteoarthritis (OA) therapy by targeting articular cartilage. OA causes enormous burden on human health and society as it is an obstinate, degradative, and complicated disease with various sequela. Compared with conventional systematic therapy that is less effective and may lead to multiple side effects, nanoparticles have been extensively exploited as novel medication to treat OA as they possess unique properties including high tissue penetration, biostability, and large specific surface area. Nano-drug delivery systems loaded with therapeutic drugs could target chondrocytes, synovium, or extracellular matrix and release the molecules sequentially. The nano therapeutical molecules could directly get to the targeted tissue, alleviating the inflammation and promoting healing. This article provides comprehensive overview of the nanomaterials targeting and treatment for OA, which could also be useful for other inflammatory diseases.

For tissue engineering, nanostructured biomaterials have shown their potent ability to enhance the regeneration of the tissue defect by actively regulate tissue forming-related cell behavior such as stem cell fate. To simulate the 3D structure of cancellous bone formation, Xia et al. prepared chitosan and hydroxyapatite nanowires (CS@HAP NWs) hybrid nanostructured scaffolds with suitable mechanical properties and porous structures. The 3D-hybrid scaffolds promote cell adhesion, migration and differentiation of human adipose-derived stem cells (hADSCs) inside the scaffolds. By rationally engineering HAP-based hybrid nanostructured scaffolds, this study provides a facile and effective approach for bone tissue engineering. In addition, titanium-based materials have been widely applied in bone-tissue engineering. However, there remains great challenge that osseointegration at the bone-implant interface is inefficient due to the inefficient neural network reconstruction. Zhao et al. used an electrochemical technique and a hydrothermal approach to construct a potassium titanate nanorod-decorated titanium oxide (K_2_Ti_6_O_13_-TiO_2_) nanotube array on the Ti implant surface. The osteogenic differentiation of mesenchymal stem cells was enhanced on the modified Ti due to the presence of nanotube array, while the neural differentiation of neural stem cells was accelerated due to the release of potassium ions from K_2_Ti_6_O_13_-TiO_2_ nanotube. The nanotube array proposed in this study is promising for promote neuralization on the surface of implants that is beneficial for bone repair.

In summary, the articles collected in this Research Topic demonstrate the development and application of biomaterials with designed nanostructures in regenerative medicine as well as disease therapy. We would like to thank the contributing authors and the reviewers for supporting this Research Topic. We believe, these articles will be helpful to researchers in both material science and biomedicine field.

